# Identification and characterization of functionally relevant SSR markers in natural *Dalbergia odorifera* populations

**DOI:** 10.1186/s12870-024-05019-2

**Published:** 2024-04-23

**Authors:** Jieru Xu, Yue Wang, Kunlin Wu, Jinhui Chen

**Affiliations:** 1 School of Breeding and Multiplication (Sanya Institute of Breeding and Multiplication), School of Tropical Agriculture and Forestry, Hainan University, Sanya, China; 2 Key Laboratory of Genetics and Germplasm Innovation of Tropical Special Forest Trees and Ornamental Plants, Ministry of Education/Engineering Research Center of Rare and Precious Tree Species in Hainan Province, School of Tropical Agriculture and Forestry, Hainan University, Haikou, China

**Keywords:** *Dalbergia odorifera*, Wood formation, SSR marker, Genetic diversity, Genetic structure

## Abstract

**Background:**

*Dalbergia odorifera* is a rare and precious rosewood specie, which is valued for its amber tones, abstract figural patterns, and impermeability to water and insects. However, the information on genetic diversity and marker-assisted selection breeding of *D. odorifera* is still limited. Simple sequence repeat (SSR) markers are an ideal tool for genetic diversity analysis and marker-assisted molecular breeding for complex traits.

**Results:**

Here, we have developed SSR markers within candidate genes and used them to explore the genetic diversity among *D. odorifera* germplasm resources. A total of 635 SSR loci were identified. The proportions of mono-, di- and tri-nucleotide repeat motifs were 52.28%, 22.99% and 21.42%, respectively. From these, a total of 114 SSR primers were synthesized, of which 24 SSR markers displayed polymorphism (polymorphic information content (*PIC*) > 0.25). Subsequently, these polymorphic markers were used for the genetic diversity analysis of 106 *D. odorifera* individuals from 11 natural populations. According to the genetic diversity analysis of *D. odorifera* natural populations, the average observed heterozygosity (*Ho*) was 0.500, the average expected heterozygosity (*He*) was 0.524, and the average Shannon’s information index (*I*) was 0.946. These indicated that the natural populations had moderate genetic diversity. AMOVA analysis showed that 5% of the total variation was within the individuals of a population, whereas 95% of the variation was among the individuals of the populations, indicating a high degree of genetic variation between populations. On the basis of their genetic structures, these populations could be divided into four groups.

**Conclusions:**

Our study provides important experimental resources for genetic studies and assists in the program of molecular breeding of *D. odorifera* wood formation.

**Supplementary Information:**

The online version contains supplementary material available at 10.1186/s12870-024-05019-2.

## Background

The genus *Dalbergia*, which belongs to the Leguminosae family, is widely distributed in tropical and subtropical regions in the world and contains many economically important species due to its timber value [1, 2]. For example, the wood of *Dalbergia odorifera* is valued for its amber tones, resistance to water and insects, pleasant aroma, and high density, which makes it as an outstanding material for making furniture and crafts. Meanwhile, its wood harbors significant medicinal properties, such as analgesic, angiogenesis and anticancer activities [3]. Most research focuses on the identification and characterization of its wood’s phytoconstituents, including flavonoids, phenols and essential oil [3]. Additionally, the analyses of population structure and genetic diversity of the specie have also been reported [4–6]. This assessment is generally a prerequisite for the genetic improvement and the breeding of new varieties.

In several cases, molecular methods, including genetic engineering, seem to be more effective than conventional breeding methods, for developing new cultivars for high yields, harboring new traits and introducing resistance against insect pests and diseases [7]. However suitable molecular tools have not yet been fully developed for *D. ororifera*. During marker-assisted selection (MAS), the use of molecular markers offers a striking opportunity for improving the phenotypic selection of desired traits for plant breeding [8]. As a result, molecular markers have gained considerable utility in genetically improving modern cultivars [9–12].

Simple sequence repeat (SSR) molecular markers, known as microsatellites or short tandem repeats (STR), consist of repeat motifs of 1–6 bp in length, and are characterized by high polymorphism that may result from different repeat numbers per SSR locus [13]. SSRs are an ideal tool to study genetic variability and perform MAS, due to the advantages of co-dominance, polymorphism, easy detection, whole-genome coverage and abundant informativeness [14, 15]. Especially, SSR molecular markers within functional genes could be more important than other genomic regions, owing to closer relationship between the expression of such genes and phenotypes of interest during the selection process [16]. Hence, the development of SSR markers within candidate genes provides a molecular basis for their potential use in MAS. SSR molecular markers also shed light on genetic diversity, genetic linkages and germplasm evaluation. For instance, rich genetic diversity of wild *Panax stipuleanatus* populations was revealed with the help of SSR markers [17]. Recently, 20 SSR markers were evaluated in 126 tea (*Camellia sinensis*) individuals from Guangxi Province and it was found that a core of six marker set had discriminated all genotypes [18]. Similarly, in *Pennisetum* population, the seven polymorphic SSR markers could be used to create molecular fingerprints of 24 cultivars [19]. Genetic diversity of 216 samples of G*inkgo biloba*, from six ancient and two cultivated populations, was investigated with the help of 22 SSR loci, which could differentiate the refuges during the glacial period from these populations [20]. Further, 40 EST-SSR markers in *Zantedeschia hybrida* were identified to create a core germplasm that included 42 individuals [21]. In addition, EST-SSR markers were developed so that they could differentiate *Opisthopappus longilobus* and *O. taihangensis* as the two distinct species [22]. Moreover, Zurn et al. (2018) constructed an 8-SSR fingerprinting set to determine 177 *Rubus* individuals from different geographic locations [23]. Meanwhile, the identification and characterization of SSR markers in previous studies are important prerequisites for plant genetic improvement.

However, compared to crops like maize and soybean, only a few studies on molecular breeding of *D. odorifera* have been reported [5]. For instance, in a previous study, 19 polymorphic SSR markers, contained in 54 alleles, among 42 samples from seven *D. odorifera* populations were developed [4]. Further, a core collection of 251 individuals, conversing the whole genetic diversity of the germplasm was evaluated with 19 SSR markers [6]. However, these studies used only a limited number of molecular markers as well as few samples to evaluate the genetic diversity of *D. odorifera*. Moreover, only a few SSR markers within candidate genes related to *D. odorifera* wood formation have been reported.

Next-generation sequencing (NGS) technology provides a tool for the development of large-scale SSR markers [24–26]. Our laboratory has previously performed RNA sequencing (RNA-seq) of specific xylem tissues of three *D. odorifera* trees and obtained candidate genes related to its xylem development [27]. Here, we mined SSR molecular markers within these candidate genes of *D. odorifera*. We obtained the polymorphic SSR markers to investigate the genetic diversity and population structure of 106 *D. odorifera* individuals. This study provides a powerful genomics tool to comprehensively analyze germplasm resources, implement an early selection of candidate traits, and improve the breeding efficiency in *D. odorifera*.

## Results

### Identification and characterization of SSR markers

**Fig. 1:**
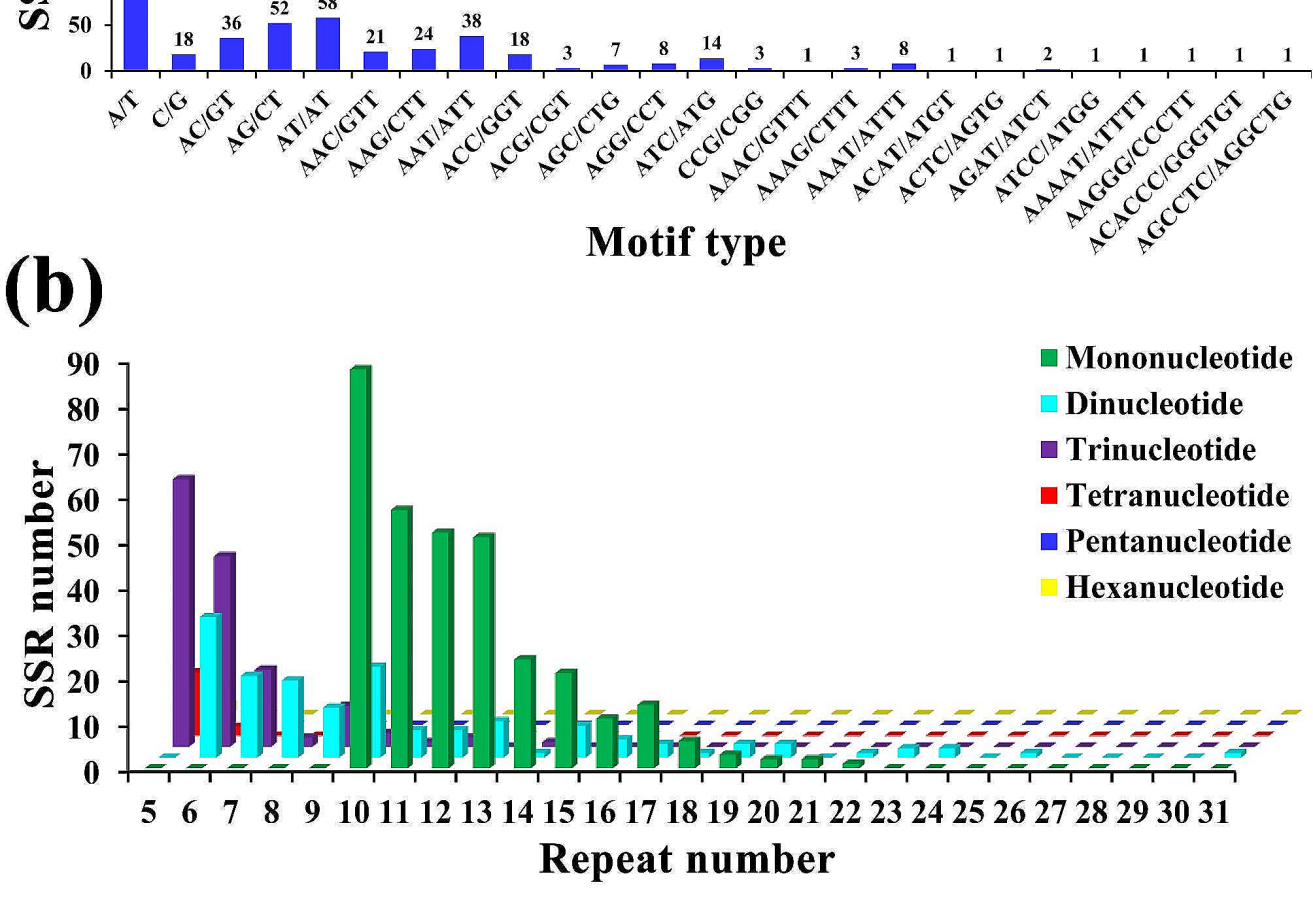
Distribution and characteristics of SSR repeat motifs within candidate genes in *Dalbergia odorifera.***a** number of motif types. **b** number of repeat motifs

We obtained 109 candidate genes related to xylem development in *D. odorifera*, which were involved in the biosynthesis of lignin, cellulose, flavonoids and terpenoids, as well as differentially expressed in xylem tissues, such as a *cinnamoyl-CoA reductase* (*CCR*) gene (evm.TU.scaffold_29.338), a *cellulose synthase A* (*CESA7*) gene (evm.TU.scaffold_39.962), a *flavonoid 3’-monooxygenase* (*CYP75B1*) gene (evm.TU.scaffold_263.278), a *cellulose synthase-like protein G2* (*CSLG2*) gene (evm.TU.scaffold_46.88), *WRKY* genes (evm.TU.scaffold_396.123 and evm.TU.scaffold_5.1084) and a *caffeoylshikimate esterase* (*CSE*) gene (evm.TU.scaffold_264.169) (Additional file 2: Table S1). They covered a cumulative of 758,570 bp, with a mean gene size of 6959 bp (Table 1). The SSR loci within these 109 genes were determined by MicroSAtellite identification tool (MISA), resulting in a total of 635 SSR loci, indicating that these genes contained abundant SSR regions (Table 1). There were 12 sequences (11.01%) that contained only one candidate SSR locus, whereas 97 sequences (88.99%) contained more than one SSR loci (Table 1). A total of 87 compound SSR loci were identified and further characterized (Table 1). On the basis of motif variation patterns, SSR loci were further classified into six different types. The mononucleotide motif was the most common (52.28%), followed by dinucleotide (22.99%) and trinucleotide (21.42%) motifs, whereas the lowest percentage was occupied by pentanucleotides (0.31%) and hexanucleotides (0.31%) (Table 1). In terms of mononucleotide repeat motifs, the most abundant type was A/T with a repeat number of at least 10, accounting for 94.58% (Fig. 1a). Among dinucleotides, the most frequent was AT/AT repeat, followed by AG/CT and AC/GT, which accounted for 39.73%, 35.62% and 24.66%, respectively (Fig. 1a). For trinucleotides, the highest percentage of repeats were AAT/ATT (27.94%), followed by AAG/CTT (17.65%) (Fig. 1a). Similarly, among the tetranucleotide motifs, the most abundant was AAAT/ATTT (47.06%) (Fig. 1a). Further, only AAAAT/ATTTT, AAGGG/CCCTT, ACACCC/GGGTGT, and AGCCTC/AGGCTG were identified as pentanucleotide and hexanucleotide repeat motifs (Fig. 1a). Generally, the repeat number varied from 5 to 31, and ten repetitions were the most for mononucleotide motifs (Fig. 1b). Moreover, repeat number of six and five was the most common for di- and trinucleotide motifs, while the repeat number more than 18 was rare (Fig. 1b).

**Table 1:** Summary of results from searching of microsatellite within candidate genes

Terms	Number
Total number of sequences	109
Total size of sequences (bp)	758,570
Total number of SSR markers	635
Total number of SSR markers containing sequences	109
Total number of sequences containing more than one SSR	97
Number of SSRs present in compound formation	87
Mononucleotide	332(52.28%)
Dinucleotide	146(22.99%)
Trinucleotide	136(21.42%)
Tetranucleotide	17(2.68%)
Pentanucleotide	2(0.31%)
Hexanucleotide	2(0.31%)

### Development of SSR primers

A total of 114 SSR loci were randomly selected to design primers, which were tested in a pilot test running on 10 DNA samples for the preliminary verification. Of these, 24 primer pairs exhibited stable and repeatable amplification, corresponding to polymorphic information content (*PIC*) > 0.25. Among these 24 SSR markers, there were 19 tri- and 5 di-nucleotide repeats (Additional file 2: Table S2). All of these 24 polymorphic SSR pairs were selected to evaluate the genetic diversity of 106 *D. odorifera* individuals.

### Polymorphism analysis of SSR loci in *D. oderifera* germplasm

The 24 SSR primers were designed from 24 gene sequences (Additional file 2: Table S2). A total of 168 different alleles were identified, and the allele number (*Na*) for each locus ranged between 2 and 16 (Table 2). The effective number of allels (*Ne*) values varied from 1.502 to 8.561, which was lower than the *Na* mean, indicating that the distribution of alleles might be out of balance (Table 2). The maximum value of the effective heterozygosity (*He*) was observed in the JXHT137 marker (Table 2), suggesting this marker possessed the highest polymorphism. The fixation index (*F*) values ranged from − 0.044 to 0.391 (Table 2), indicating that the number of homozygotes was higher than heterozygotes among the assayed populations.

**Table 2:** Polymorphism characteristics of 24 SSR loci in 106 *Dalbergia odorifera* individuals

Locus	*Na*	*Ne*	*I*	*Ho*	*He*	*F*	*PIC*
JXHT002	5	1.647	0.835	0.330	0.393	0.160	0.374
JXHT004	5	2.226	1.071	0.481	0.551	0.127	0.514
JXHT005	7	1.657	0.849	0.340	0.396	0.143	0.373
JXHT010	6	1.502	0.714	0.340	0.334	-0.016	0.314
JXHT013	3	1.938	0.831	0.377	0.484	0.220	0.427
JXHT022	2	1.588	0.557	0.377	0.370	-0.019	0.302
JXHT025	5	2.190	0.903	0.434	0.543	0.202	0.444
JXHT034	3	2.067	0.771	0.481	0.516	0.068	0.401
JXHT051	4	2.946	1.134	0.538	0.661	0.186	0.590
JXHT062	4	1.907	0.776	0.406	0.476	0.147	0.390
JXHT066	6	2.852	1.196	0.538	0.649	0.172	0.589
JXHT081	3	2.063	0.813	0.453	0.515	0.121	0.419
JXHT094	5	2.635	1.085	0.585	0.621	0.057	0.548
JXHT097	7	3.840	1.429	0.689	0.740	0.069	0.693
JXHT098	7	2.669	1.248	0.610	0.625	0.025	0.576
JXHT100	6	1.814	0.863	0.340	0.449	0.243	0.404
JXHT104	5	2.311	1.040	0.495	0.567	0.127	0.497
JXHT105	13	5.721	1.911	0.726	0.825	0.120	0.802
JXHT121	13	2.657	1.426	0.514	0.624	0.175	0.590
JXHT129	5	3.129	1.241	0.660	0.680	0.029	0.620
JXHT130	11	3.405	1.622	0.635	0.706	0.102	0.681
JXHT133	11	4.275	1.642	0.726	0.766	0.052	0.729
JXHT136	11	6.450	2.022	0.882	0.845	-0.044	0.827
JXHT137	16	8.561	2.319	0.538	0.883	0.391	0.872
Mean	7	3.002	1.179	0.521	0.593	0.119	0.541

Note: *Na*: observed number of alleles; *Ne*: effective number of alleles; *I*: Shannon’s information index; *Ho*: observed heterozygosity; *He*: expected heterozygosity; *F*: fixation index; *PIC*: polymorphism information content

The polymorphism level of a locus could be assessed by* PIC*. Generally, when *PIC* < 0.25, it represents low polymorphism, 0.25–0.5 indicates moderate polymorphism, and *PIC* > 0.5 shows high polymorphism [28]. A total of 13 loci, namely JXHT004, JXHT051, JXHT066, JXHT094, JXHT097, JXHT098, JXHT105, JXHT121, JXHT129, JXHT130, JXHT133, JXHT136 and JXHT137, showed high polymorphism (*PIC* values between 0.514 and 0.872; Table 2). In contrast, the lowest *PIC* value (0.302) was observed for the JXHT022 locus (Table 2). In general, the JXHT137 locus showed the highest diversity, as this locus had the biggest *Na* (16), *Ne* (8.561), Shannon’s information index (*I*, 2.319), observed heterozygosity (*Ho*, 0.538), *He* (0.883), and *PIC* (0.872) values (Table 2).

### Hierarchical cluster analysis of 106 *D. odorifera* individuals

Cluster analysis of 106 *D. odorifera* individuals was performed on the basis of their Nei’s genetic distances. These individuals were separated into 14 major groups (represented with different colors in Additional file 1: Fig. S1). B049 and B097, from Dongfang and Tunchang, respectively, were grouped in Cluster I (Additional file 1: Fig. S1). B089, from Qionghai, was the only member of Cluster II (Additional file 1: Fig. S1). Cluster III consisted of two individuals (B078 and B095) from two different regions (Additional file 1: Fig. S1). Moreover, the genetic distance between the individuals of clusters I-III and the remaining were the largest (Additional file 1: Fig. S1), indicating that these individuals may have derived from the most primitive *D. odorifera* germplasm resources. The majority of *D. odorifera* individuals were included in Cluster XIV (Additional file 1: Fig. S1), which showed a close relationship among them. Interestingly, the correlation between genetic distance and geographical distance might not be significant for these 106 *D. odorifera* individuals.

### Genetic diversity of *D. odorifera*

On the basis of their geographic locations, 106 individuals of *D. odorifera* could be grouped into 11 populations (Additional file 1: Fig. S2; Table 3). To evaluate the genetic diversity, we calculated the values of *Na*, *Ne*, *I*, *Ho*, *He* and *F* of these populations with the help of GenALEx software. At the population level, the average value of *Ho* (0.500) was lower than *He* (0.524), suggesting a lack of heterozygotes among these populations (Table 4). The *F* values ranged from − 0.105 to 0.136 (mean = 0.034), where negative values were obtained for the populations 2, 8 and 11, which implied a high diversity and heterozygosity in these three populations (Table 4). Additionally, the *I*-values varied from 0.583 to 1.146 (average, 0.946; Table 4). Generally, the natural populations of *D. odorifera* showed moderate levels of genetic diversity. Amongst them, population 1 (Haikou and Ding’an) showed the highest genetic diversity, with the highest values for *Na* (5), *Ne* (2.983), *I* (1.146) and *F* (0.136), while population 6 (Wenchang) showed the lowest genetic diversity, as inferred from the smallest values of *Na* (2), *Ne* (1.775), *I* (0.583), *Ho* (0.319) and *He* (0.375) (Table 4).

**Table 3:** Geographical location of 106 *Dalbergia odorifera* individuals

Population	Locality	Code	E longitude	N latitude	Number
1	Haikou	B001-B028	110°13′27″-110°32′06″	19°42′27″-20°02′23″	28
	Ding’an	B100-B101	110°12′03″-110°14′29″	19°36′12″-19°38′56″	2
2	Danzhou	B029-B042	109°13′28″-109°40′15″	19°27′59″-19°47′34″	14
3	Dongfang	B043-B049	108°40′36″-108°47′40″	18°48′11″-19°06′59″	7
4	Ledong	B050-B058	108°42′39″-108°57′35″	18°27′34″-18°41′46″	9
5	Sanya	B059-B072	109°09′43″-109°32′56″	18°13′34″-18°22′35″	14
6	Wenchang	B073-B075	110°44′48″-110°54′51″	19°32′37″-19°34′56″	3
7	Wanning	B076-B083	110°13′12″-110°29′02″	18°47′52″-19°01′16″	8
	Qionghai	B089-B090	110°24′23″-110°28′58″	19°07′56″-19°15′34″	2
8	Chengmai	B084-B086	110°00′50″-110°06′41″	19°44′41″-19°55′37″	3
9	Lingao	B091-B096	109°33′50″-109°41′00″	19°54′43″-19°56′44″	6
10	Tunchuang	B097-B099	109°59′04″-110°06′36″	19°13′14″-19°21′26″	3
11	Baoting	B087-B088	109°42′42″-109°48′40″	18°38′41″-18°39′18″	2
	Lingshui	B102	110°02′38″	18°30′44.39″	1
	Baisha	B103-B104	109°16′38″-109°26′57″	19°09′39″-19°13′40″	2
	Changjiang	B105	109°05′23″	19°07′39″	1
	Wuzhishan	B106	110°23′26″	19°59′26″	1

**Table 4:** Genetic diversity analysis of *Dalbergia odorifera* populations

Population	*N*	*Na*	*Ne*	*I*	*Ho*	*He*	*F*
1	30	5	2.983	1.146	0.503	0.587	0.136
2	14	4	2.488	0.979	0.583	0.525	-0.105
3	7	4	2.503	1.011	0.535	0.569	0.044
4	9	4	2.625	1.019	0.519	0.564	0.070
5	14	4	2.873	1.091	0.553	0.573	0.025
6	3	2	1.775	0.583	0.319	0.375	0.118
7	10	4	2.848	1.067	0.514	0.573	0.084
8	3	3	2.215	0.709	0.444	0.417	-0.070
9	6	3	2.145	0.795	0.449	0.460	0.022
10	3	3	2.420	0.898	0.486	0.525	0.060
11	7	4	2.907	1.111	0.595	0.592	-0.014
Mean	10	4	2.526	0.946	0.500	0.524	0.034

Note: *N*: number of individuals per population; *Na*: observed number of alleles; *Ne*: effective number of alleles; *I*: Shannon’s information index; *Ho*: observed heterozygosity; *He*: expected heterozygosity; *F*: fixation index

### Genetic structure and differentiation analysis

The molecular variation across 11 populations was analyzed with the help of the analysis of molecular variance (AMOVA). Only 5% of the total variation was within the individuals of a population, whereas 95% of the variation was among the individuals from different populations (Additional file 2: Table S3). The genetic variation among the populations was abundant than within the population, accounting for the main variation. The genetic differentiation index (*F*_*ST*_) values between all pairs varied from 0.010 to 0.125 (Additional file 2: Table S4), revealing that little genetic variation existed among the populations. The *F*_*ST*_ value between populations 6 and 10 was the maximum, whereas the value between populations 1 and 5 was the minimum (Additional file 2: Table S4).

To deeply explore the genetic structure, analysis of genetic distance and genetic identity were performed, as shown in Additional file 1: Fig. S3. Genetic distances between the Tunchang population and others were the highest (Additional file 1: Fig. S3a), whereas the genetic identity (between the Tunchang and other populations) was lowest (Additional file 1: Fig. S3b), which indicated lowest similarity.

**Fig. 2:**
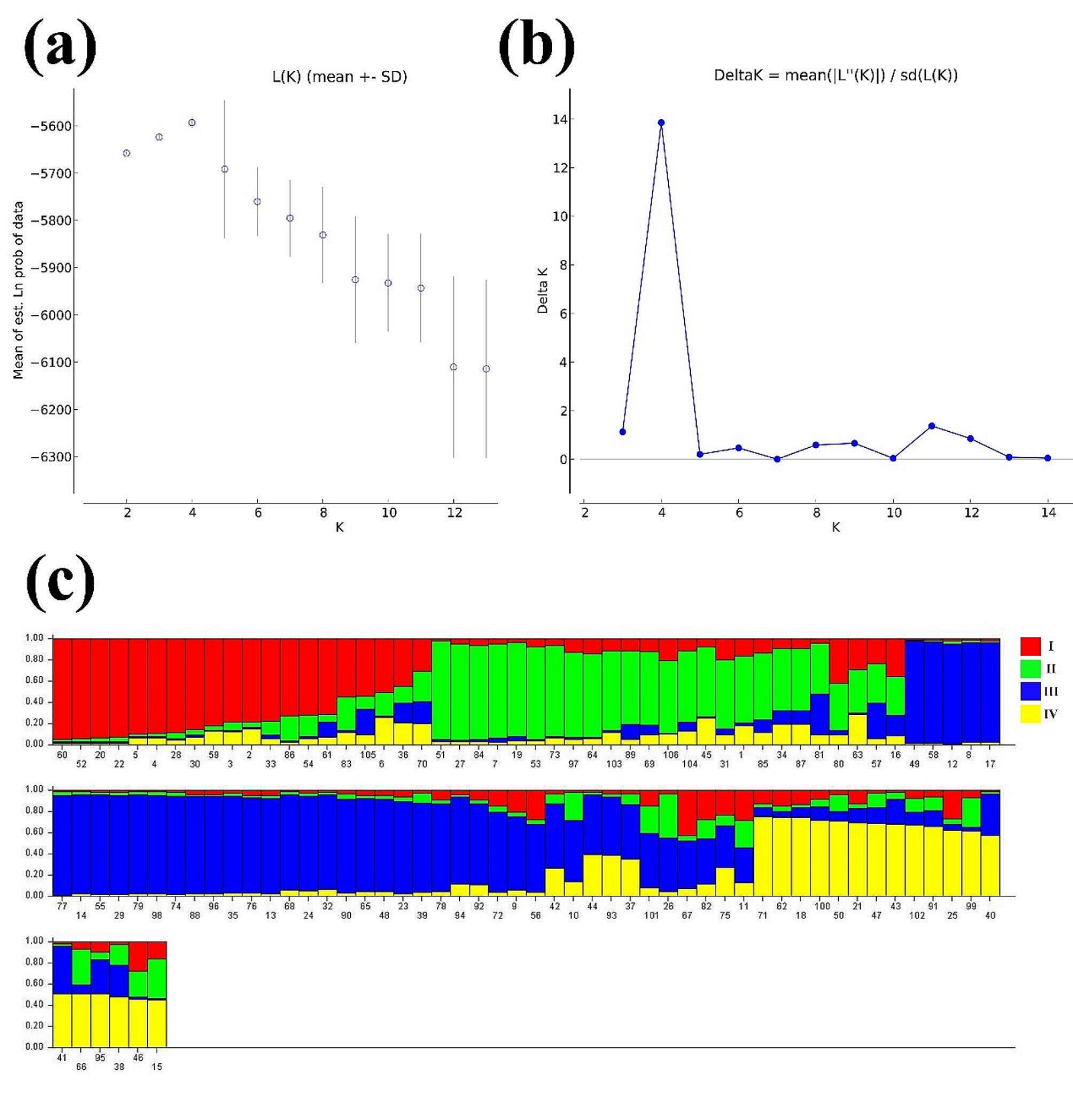
Structure analysis of 106 *Dalbergia odorifera* individuals. **a** LnP(D) values for each population number (K). **b** Relationships between the K values and the corresponding ΔK statistics. **c** Detailed bar plots of 106 individuals of *D. odorifera* analyzed by Structure software based on 24 SSR markers (K = 4). The horizontal axis represents a single individual and different colors indicate different groups. The vertical line represents admixture in an individual and the proportion of its genome in K groups

The population structure of 106 individuals of 11 populations was defined using the Structure software. The peak of the lnP(D) values was observed at K = 4 (Fig. 2a). Meanwhile, according to ΔK values, when K was 4, ΔK reached the peak of 13.86 (Fig. 2b). These results demonstrated that the genetic information of 106 individuals, from 11 populations, could be further assigned to four different genetic groups. Group IV contained the majority of individuals (yellow), followed by groups III (blue) and II (green) (Fig. 2c). Interestingly, we found that there was no clear relationship between the genetic relationships and geographic origin of 106 individuals.

### Cross amplification of SSR markers to other species

Four species, including three *Dalbergia tonkinensis* Prain. individuals, three *D. sissoo* individuals, one *D. cochinchinensis* individual and three *Pterocarpus santalinus* individuals, were evaluated with 24 markers for the SSR transferability. The results showed that DNA fragments were generated by 24 SSR markers in *D. tonkinensis* (Table 5). However, two and six markers failed to yield amplicons in the *D. sisso* and *D. cochinchinensis* individual, respectively, including JXHT098, JXHT105, JXHT002, JXHT005, JXHT022, JXHT062, JXHT066 (Table 5). In *P. santalinus* individuals, 12 out of 24 SSR markers could be successfully amplified (Table 5).

**Table 5:** Transferability of 24 primer pairs in *Dalbergia* and *Pterocarpus* species

Locus	*Dalbergia tonkinensis*	*Dalbergia cochinchinensis*	*Dalbergia sissoo*	*Pterocarpus santalinus*
JXHT002	**+**	**-**	**+**	**-**
JXHT004	**+**	**+**	**+**	**+**
JXHT005	**+**	**-**	**+**	**-**
JXHT010	**+**	**+**	**+**	**+**
JXHT013	**+**	**+**	**+**	**-**
JXHT022	**+**	**-**	**+**	**+**
JXHT025	**+**	**+**	**+**	**-**
JXHT034	**+**	**+**	**+**	**+**
JXHT051	**+**	**+**	**+**	**+**
JXHT062	**+**	**-**	**+**	**+**
JXHT066	**+**	**-**	**+**	**-**
JXHT081	**+**	**+**	**+**	**-**
JXHT094	**+**	**+**	**+**	**-**
JXHT097	**+**	**+**	**+**	**+**
JXHT098	**+**	**+**	**-**	**-**
JXHT100	**+**	**+**	**+**	**+**
JXHT104	**+**	**+**	**+**	**+**
JXHT105	**+**	**-**	**-**	**-**
JXHT121	**+**	**+**	**+**	**+**
JXHT129	**+**	**+**	**+**	**+**
JXHT130	**+**	**+**	**+**	**+**
JXHT133	**+**	**+**	**+**	**-**
JXHT136	**+**	**+**	**+**	**-**
JXHT137	**+**	**+**	**+**	**-**

*Note* “+” shows all tested loci were successfully transferred, while “-” shows none SSR loci amplified

A unweighted pair group method using arithmetic averages (UPGMA) dendrogram of 30 individuals from five species, including *D. odorifera* (B007, B009, B012, B018, B020, B023, B029, B033, B038, B039, B046, B047, B048, B051, B053, B054, B060, B058, B070 and B069), *D. tonkinensis* (B108-B110), *D. sissoo* (B112-B114), *D. cochinchinensis* (B111) and *P. santalinus* (B115-B117), was constructed to evaluate the genetic relationship. These 30 individuals could be grouped into the major five clusters (Additional file 1: Fig. S4a). Cluster I, II, III, IV and V corresponded to individuals from *D. odorifera*, *D. tonkinensis*, *D. sissoo*, *P. santalinus and D. cochinchinensis*, respectively (Additional file 1: Fig. S4a), showing the obvious separation between the five species. *D. odorifera* showed the closest genetic relationship with *D. tonkinensis*, while it could be farthest from *D. cochinchinensis* (Additional file 1: Fig. S4a). Additionally, results of a principal coordinate analysis (PCoA) supported those from cluster analysis as it distinguished all species into five groups (Additional file 1: Fig. S4b). The first two principal coordinate accounted for 40.83% of the total variation (Additional file 1: Fig. S4b).

## Discussion

### Development of SSR markers from candidate genes to improve plant breeding studies

MAS strongly assists in phenotypic selections, where the inclusion of molecular markers offers a striking promise for plant breeding [8]. For instance, in *Castanea sativa*, the FIR059 locus could discriminate drought-susceptible and drought-tolerant individuals and was applied to select drought-tolerant *C. sativa* trees [29]. Two EST-SSR markers were mined by using association mapping within the target quantitative trait loci (QTL), which supported sugar yield in sugarcane [11]. Based on *Prunus dulcis* RNA-sequencing, several SSR loci within candidate genes, in response to abiotic stresses, were identified. These markers investigated the genetic diversity and population structure of genotypes from *P. dulcis* and its related species [30]. The studies above offer an informative framework that could be applicable to studies on the use of SSR markers for genetic diversity and MAS of *D. odorifera*. Meanwhile, the recently published genome of *D. odorifera* genome [31] provides a basic resource for the development of molecular markers.

In this study, we have developed SSR markers within functional genes that are related to wood formation in *D. odorifera*. These SSR markers are useful for accurate individual identification and confirmed plant parentage. Based on our present study, the identity of *D. odorifera* would be verified using our SSR markers, which will help in determining the parentage of unknown or doubtful *D. odorifera*, establishing *D. odorifera* plantations, and conserving *D. odorifera* genetic resources. Furthermore, our future work will focus on marker–trait associations and finally acquire candidate markers with the goal of enhancing the quality and quantity of wood products. The elite *D. odorifera* would be selected for mating systems by SSR markers, resulting in the progeny harboring different combinations of beneficial wood–quality traits. In addition, the markers would be useful for early identification of elite *D. odorifera* trees, aimed at increasing the efficiency and effectiveness of genetic improvement.

Additionally, SSR expansion or contraction directly affects the corresponding gene products and could even cause phenotypic changes [16]. Hence, variations in SSR repeat numbers within genes are critical for normal gene activity [16]. Further, SSR variations within coding regions may lead to the gain or loss of gene function or gene silencing [16]. SSR variations in 5’-UTRs, 3’-UTRs and introns may result in a loss of gene function by mRNA splicing or inhibition of translation [16]. Therefore, the development of SSR markers and the characterization of their polymorphism among germplasm resources are essential prerequisites for their application in MAS of important economic traits. Further, we have attempted to mine SSR markers that might be significantly linked to wood traits, and have provided a molecular genetic basis for an early trait selection and accelerating the breeding efficiency.

### Characterization of SSR markers in *D. odorifera*

A total of 24 SSR loci (21.05%) showed moderate and high polymorphism (*PIC* > 0.25) (Table 2). The proportion of polymorphic primers was much lower than that observed in other species. For instance, 41.1% of primers were polymorphic in *Ricinus communis* [32], 50% were reported in *Parrotia subaequalis* [33], and 41.1% were reported in *Lycopersicon esculentum* [34]. On the other hand, 21.4% polymorphic primers were reported in *P. notoginseng* [17].

These 24 SSR markers were analyzed in 106 individuals from 11 populations of *D. odorifera*. We found that 13 of these SSR loci were highly polymorphic (*PIC* > 0.5), whereas the others showed moderate polymorphism (*PIC* values between 0.302 and 0.497; Table 2). The results were similar to those of Su et al. (2023), who had found that 14 of 67 preliminarily selected EST-SSR primers were polymorphic when they were tested on 48 *Elymus nutans* genotypes [17]. Moreover, these 24 SSR markers seem to be cost-effective for studying species related to *D. odorifera*, as these markers could be applied to detect variability in *D. tonkinensis*, *D. sissoo*, *D. cochinchinensis*, and *P. santalinus* (Table 5). We infer that these SSR markers could be useful tools for further genetic diversity analysis for the related species of *D. odorifera*. In addition, the transferable feature of the SSR markers between related species expands their utility in plant genetic studies [24, 30, 35, 36]. In summary, the SSR markers developed in this study display moderate and high polymorphism, and might be an excellent tool for investigating genetic relationships, varietal identity and genetic diversity analysis.

### Genetic diversity in *D. odorifera* natural populations

*D. odorifera*, an endemic to Hainan Island, is renowned for its heartwood. But its wild populations have been dramatically destroyed due to its over-exploitation. Recently, some studies have started to explore the genetic diversity of this species, and provide useful information for resource conservation and breeding strategies [4]. These studies, however, have failed to provide a unified sample that represents the genetic diversity of *D. odorifera* germplasm in Hainan Province. Clearly, information on the collective genetic diversity of this species across Hainan Island is lacking. Moreover, the molecular markers developed in previous studies have lacked reference to the *D. odorifera* genome. Therefore, in this study, we collected 106 individuals from 17 regions that represent all the *D. odorifera* natural populations. Further, we investigated the genetic diversity and population structure of these individuals using SSR molecular markers, which we developed by exploiting genome-wide information.

*D. odorifera* natural populations had medium levels of genetic diversity, with an average observed *Ho* of 0.500, *He* of 0.524, and *I* of 0.946 (Table 4). Cluster analysis indicated that these individuals were split into four main groups (K = 4), with an admixture model regardless of geographical distribution (Fig. 2c). Molecular variation analysis indicated that only 5% diversity existed within the individuals of a population, while among the individuals of the populations, it was 95% (Additional file 2: Table S3), which was similar to the reports of Liu et al. [6]. Additionally, based on Nei’s genetic distance, these 106 individuals were clustered into 14 main groups, irrespective of their populations and geographical distribution (Additional file 1: Fig. S1), thus providing insights into *D. odorifera* introduction and spread across Hainan Island.

## Conclusions

Here, we have provided a set of 24 polymorphic SSR molecular markers, developed within functional genes that are related to wood formation in *D. odorifera*. Using these markers, we have elucidated the genetic diversity and population structure of *D. odorifera* germplasm resources. Based on genetic diversity analysis, the average values of *Ho*, *He* and *I* were 0.500, 0.524 and 0.946, respectively, indicating that the natural populations had moderate genetic diversity. Based on AMOVA analysis, we infer that most of the genetic variation existed among the individuals of the populations (95%) rather than within the individuals of a population (5%). Further, the 106 *D. odorifera* individuals could be grouped into four groups. Moreover, the 24 SSR markers are cost-effective, and could be applied to study the related species of *D. odorifera.* The candidate gene-based SSR markers obtained in this study would be highly effective in association studies, traits dissection and molecular breeding projects of *D. odorifera*.

## Methods

### Identification of microsatellite loci from *D. Odorifera* transcriptome

The functional genes related to wood formation in *D. odorifera* were obtained from *D. odorifera* transcriptome data [27]. These genes were involved in the biosynthesis of lignin, cellulose, flavonoids and terpenoids, and were highly expressed in xylem tissues (Additional file 2: Table S1). The SSR identification within these gene sequences was performed with the help of the MISA software (https://webblast.ipk-gatersleben.de/misa/). As criteria, the minimum repeat numbers of mono-, di, tri-, tetra, penta-, and hexanucleotide motifs were 10, 6, 5, 5, 5, and 5, respectively. Tri-, tetra-, penta-, and hexa-nucleotide motif types were retrieved and SSR loci containing only C/G were discarded. Subsequently, the primer pairs of the rest of the SSR markers were designed with the help of Primer v5 software with following parameters; length of 18–25 bp, melting temperature (Tm) between 56 and 62 °C (the temperature difference between one primer pair within 3 °C), PCR products of 100–300 bp, the GC content of 50-60%, and within 4 consecutive paired bases for one primer pair.

### Plant materials and DNA extraction

A total of 106 *D. odorifera* individuals (B001-B106) were used to study the genetic diversity. These individuals, from 17 regions, were attributed to 11 populations on the basis of their geographical locations (Table 3). Around two leaves of each individual were collected for DNA extraction. Meanwhile, seven individuals of its related species were also collected, which included *D. tonkinensis.*, *D. sissoo*, and *D. cochinchinensis*. Also, *P. santalinus* was used to analyze SSR marker transferability. We used an RNAprep pure plant plus kit (Tiangen, Beijing, China) to extract the genomic DNA. The good quality of DNA (1.9 ≤ A260/A280 ≤ 2.2, A260/A230 ≥ 2.0) was saved in our laboratory for future use.

### Verification of polymorphism of SSR markers

We randomly selected 114 SSR primer pairs for their synthesis. First, a pilot experiment was conducted where these SSR primers were used to amplify 10 DNA samples. PCR amplification for all markers was conducted in a 15 µL volume, including 7.5 µL of 2×Taq PCR master mix, 2.0 µL of specific primer pairs, 1.0 µL of DNA template, and 4.5 µL of ddH_2_O. The PCR conditions were as follows: pre-denaturation at 96 °C for 3 min, 96 °C for 30 s, annealing at 56–62 °C for 30 s (depending on the primers), 30 cycles at 72 °C for 1 min, and a final extension at 72 °C for 10 min. The PCR products were examined on an ABI 3730xl DNA analyzer, and the results were evaluated by GeneMarker v2.2.0 to determine the polymorphism and genetic diversity in 106 *D. odorifera* individuals (our data were deposited at the Genome Sequence Archive in BIG Data Center (BIG Data Center Members, 2019), Beijing Institute of Genomics (BIG), Chinese Academy of Sciences under accession number OMIX005222).

### Statistical analysis

Statistical analysis for each SSR primer pair was conducted with the help of GenALEx [37]. *Na*, *Ne*, *I*, *Ho*, *He*, *F*, *F*_*st*_ were calculated. *PIC* was calculated by using the Cervus software [38]. Additionally, the genetic structure of *D. odorifera* populations was determined by Structure v2.3.4 based on admixture’ model [39]. The analysis was running 20 independent times for the population numbers (K) ranging from 2 to 15, with a burn-in of 10,000 iterations, and 100,000 subsequent MCMC steps were adapted in utilizing the Structure software [40]. The optimal △K was determined to evaluate K by STRUCTURE HARVESTER (https://taylor0.biology.ucla.edu/structureHarvester/).

Cluster analysis of 106 individuals was performed on the basis of Nei’s genetic distance, with the help of the poppr package in R-studio. UPGMA based cluster analysis of *D. odorifera* and its relatives was conducted with the help of the NTsys software (Applied Biostatistics Inc Setauket, USA). The genetic variation among and within the populations was investigated with AMOVA by using GenALEx. Further, the PCoA, based on Nei’s genetic distance, was also performed by GenALEx.

### Electronic supplementary material

Below is the link to the electronic supplementary material.

Supplementary Material 1

## Data Availability

We obtained sampling permission before sampling our experiment materials, and our data were deposited at the Genome Sequence Archive in BIG Data Center (BIG Data Center Members, 2019), Beijing Institute of Genomics (BIG), Chinese Academy of Sciences under accession number OMIX005222.
