# Supplementary material for: Identification and characterization of functionally relevant SSR markers in natural Dalbergia odorifera populations
Source: BMC Plant Biol. 2024 Apr 23;24:315. doi: 10.1186/s12870-024-05019-2 (PMC11036651; doi:10.1186/s12870-024-05019-2)
Supplement: Supplementary file 1 — Supplementary Material 1 [file 12870_2024_5019_MOESM1_ESM.pdf]

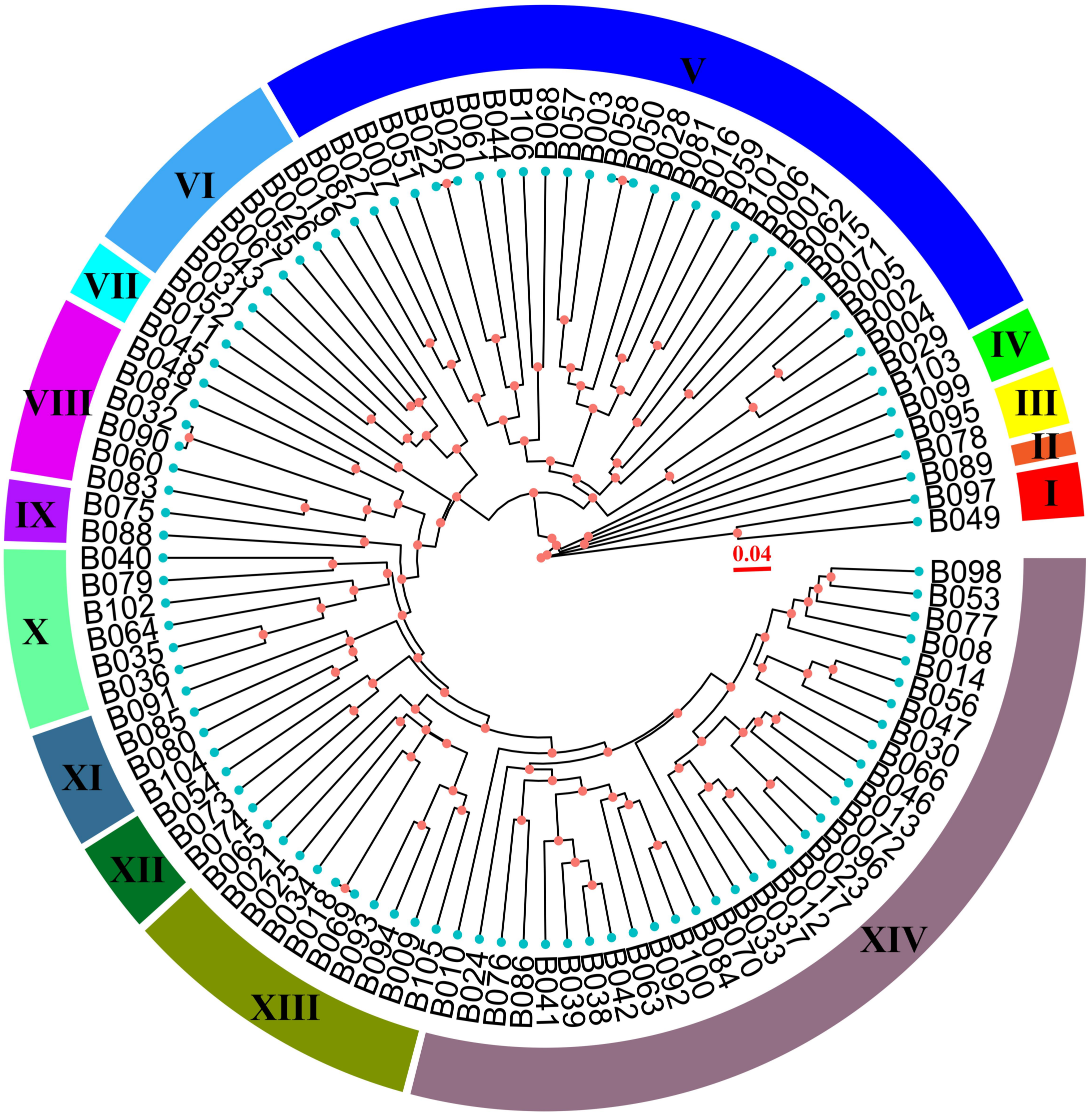


**Fig. S1** Cluster analysis of 106 *Dalbergia odorifera* individuals on the basis of Nei's genetic distance.

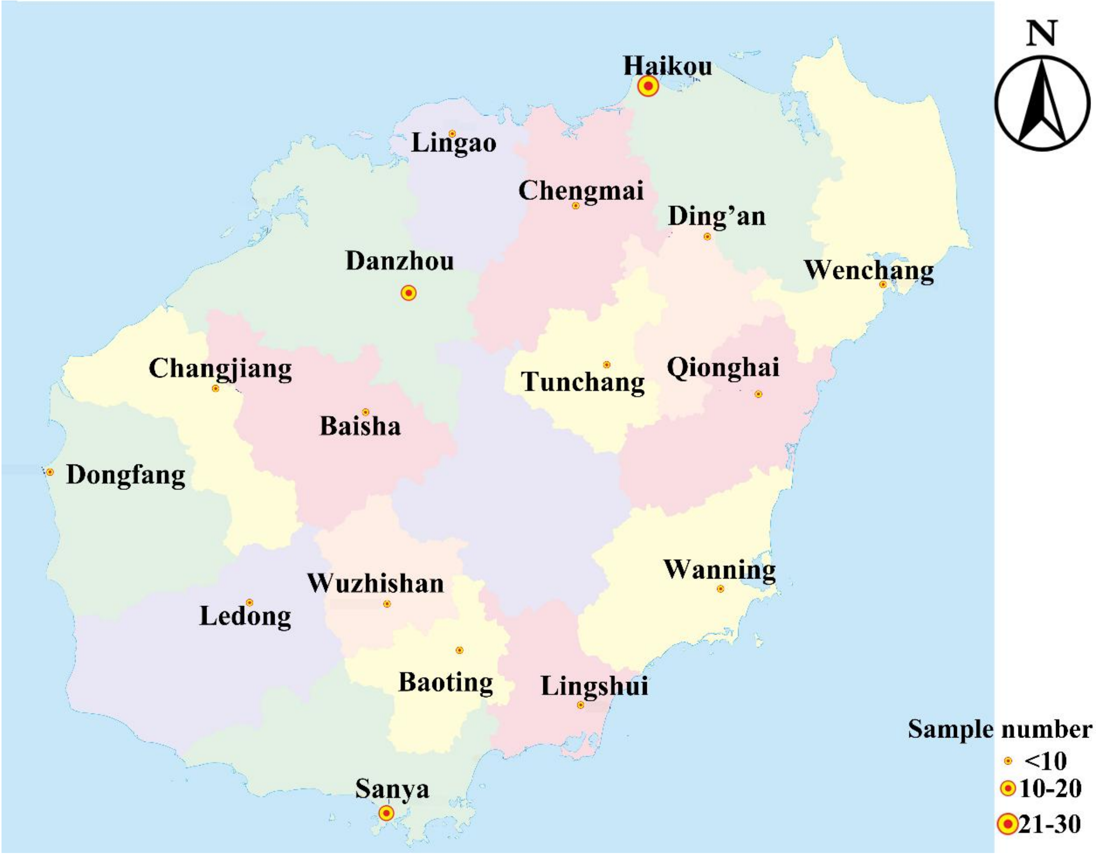


**Fig. S2** Geographic distribution of 106 *Dalbergia odorifera* individuals in Hainan Island, see Table 3 for detail. The circles in different diameter represented the sample number. Figure number: Qiong S (2024) 036.

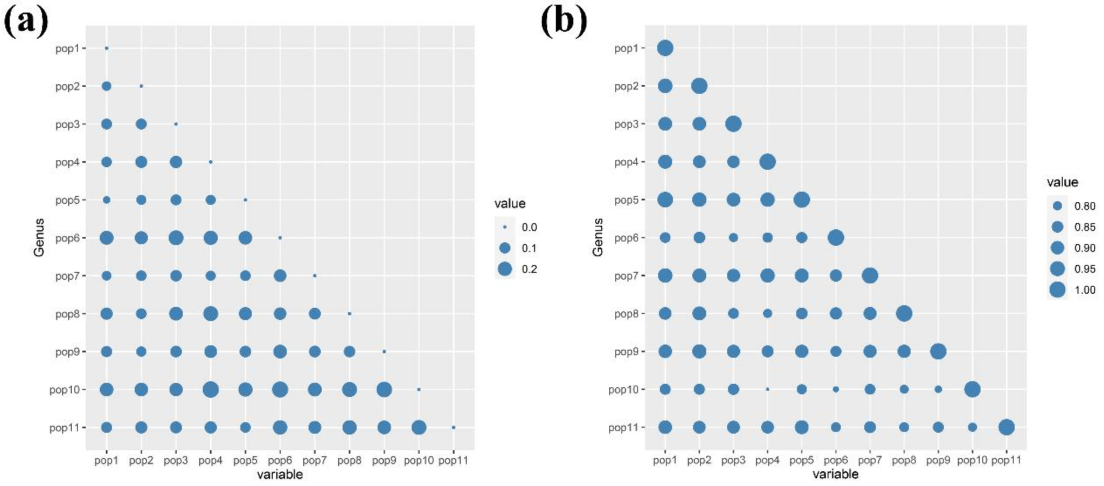


**Fig. S3** Nei’s genetic distance values and genetic identity values between 11 populations of *Dalbergia odorifera.* **a** Genetic distance. **b** genetic identity.

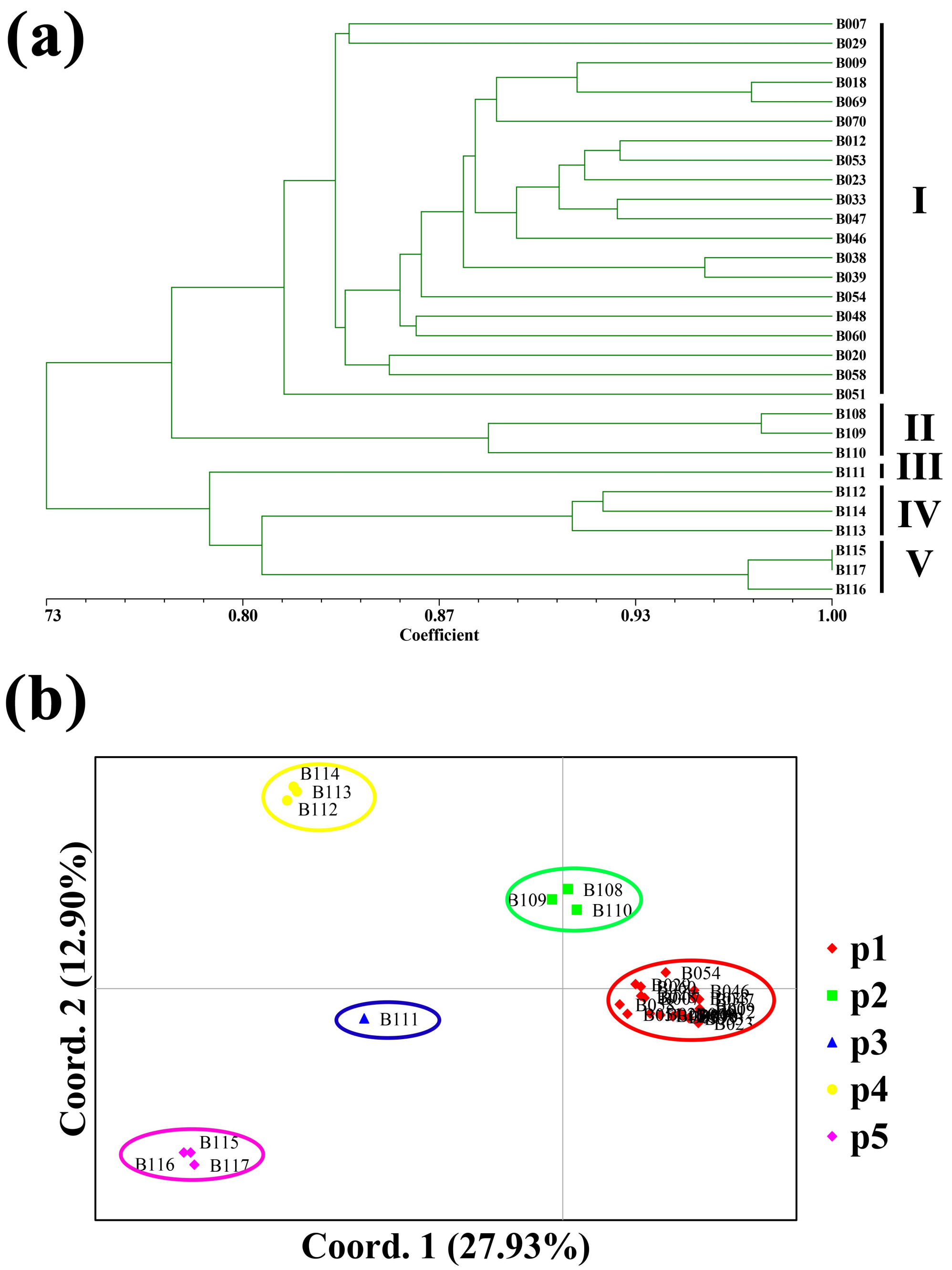


**Fig. S4** UPGMA dendrogram and principal component plot. **a** UPGMA dendrogram of 30 individuals of *Dalbergia odorifera*, *Dalbergia tonkinensis*, *Dalbergia sissoo*, *Dalbergia cochinchinensis*, and *Pterocarpus santalinus*, produced by NTsys software. **b** The principal coordinates analysis (PCoA) for these five species by GenALEx. Results of both the types of analyses showed that these individuals could be split into five clusters.

**Table S1** Candidate genes used for the SSR marker development

| Gene id | Putative function based on sequence homology | Size/bp |
|---|---|---|
| evm.TU.scaffold_100.36 | GDP-mannose 4,6 dehydratase 1 | 1479 |
| evm.TU.scaffold_100.115 | Transmembrane protein 53-B | 4506 |
| evm.TU.scaffold_100.566 | DeSI-like protein At4g17486 | 4334 |
| evm.TU.scaffold_29.376 | Protein ORANGE | 3732 |
| evm.TU.scaffold_206.373 | Protein breast cancer susceptibility 1 homolog | 11663 |
| evm.TU.scaffold_320.176 | Urease accessory protein UreH | 1915 |
| evm.TU.scaffold_248.80_evm .TU.scaffold_248.81 | NF-X1-type zinc finger protein NFXL2 | 6964 |
| evm.TU.scaffold_5.1493 | 50S ribosomal protein L21 | 3331 |
| evm.TU.scaffold_5.496 | Calpain-type cysteine protease DEK1 | 18614 |
| evm.TU.scaffold_5.194 | Ras-related protein RABC1 | 3492 |
| evm.TU.scaffold_35.178 | La-related protein 1C | 4344 |
| evm.TU.scaffold_35.233 | Probable calcium-binding protein CML21 | 3782 |
| evm.TU.scaffold_461.106 | Mediator of RNA polymerase II transcription subunit 17 | 4244 |
| evm.TU.scaffold_35.946 | Transcription initiation factor TFIID subunit 6 | 12534 |
| evm.TU.scaffold_375.165 | Elongator complex protein 1 | 5542 |
| evm.TU.scaffold_416.88 | Threonine dehydratase biosynthetic | 4934 |
| evm.TU.scaffold_416.207 | Probable CCR4-associated factor 1 homolog 9 | 873 |
| evm.TU.scaffold_111.172 | Red chlorophyll catabolite reductase | 2183 |
| evm.TU.scaffold_97.228 | MAU2 chromatid cohesion factor homolog | 9995 |
| evm.TU.scaffold_37.93 | Probable monogalactosyldiacylglycerol synthase | 9854 |
| evm.TU.scaffold_36.48 | F-box protein SKP2B | 1090 |
| evm.TU.scaffold_205.141 | Pyrophosphate--fructose 6-phosphate 1-phosphotransferase subunit alpha | 5914 |
| evm.TU.scaffold_40.997 | Protein enhanced disease resistance 2-like | 7647 |
| evm.TU.scaffold_14.222 | ABC transporter B family member 20 | 10658 |
| evm.TU.scaffold_273.148 | Kinesin-like protein KIN-UB | 16937 |
| evm.TU.scaffold_350.54 | UDP-glucose flavonoid 3-O-glucosyltransferase 7 | 1720 |
| evm.TU.scaffold_233.143 | Apyrase 2 | 7339 |
| evm.TU.scaffold_222.918 | Uncharacterized | 3660 |
| evm.TU.scaffold_99.48 | Subtilisin-like protease SBT1.5 | 3677 |
| evm.TU.scaffold_206.836 | Pentatricopeptide repeat-containing protein At1g73710 | 3210 |
| evm.TU.scaffold_145.62 | Coiled-coil domain-containing protein SCD2 | 14399 |
| evm.TU.scaffold_263.147 | Protein CHUP1 | 5608 |
| evm.TU.scaffold_46.329 | Metal-nicotianamine transporter YSL1 | 3705 |
| evm.TU.scaffold_416.30 | Splicing factor 3A subunit 2 | 7229 |
| evm.TU.scaffold_376.165 | TITAN-like protein | 3777 |
| evm.TU.scaffold_396.123 | WRKY transcription factor 22 | 2272 |
| evm.TU.scaffold_222.981 | Cytochrome P450 94C1 | 2332 |
| evm.TU.scaffold_14.215 | Probable sulfate transporter 4.2 | 7831 |
| evm.TU.scaffold_222.700 | UDP-glucuronic acid decarboxylase 2 | 5798 |
| Novelgene1059 | Probable aldo-keto reductase 1 | 15951 |

| | | |
| --- | --- | --- |
| evm.TU.scaffold_100.620 | Adenine phosphoribosyltransferase 3 | 4626 |
| evm.TU.scaffold_100.642 | UDP-glucuronate 4-epimerase 3 | 2991 |
| evm.TU.scaffold_100.921 | Cellulose synthase-like protein D3 | 5485 |
| evm.TU.scaffold_185.56 | Phospholipase D delta | 28455 |
| evm.TU.scaffold_29.338 | Cinnamoyl-CoA reductase 1 | 12949 |
| evm.TU.scaffold_29.681 | Beta-carotene hydroxylase 2 | 2515 |
| evm.TU.scaffold_100.76 | Protein shi related sequence 1 | 2972 |
| evm.TU.scaffold_39.265 | Topless-related protein 2 | 9260 |
| evm.TU.scaffold_39.962 | Cellulose synthase A catalytic subunit 7 | 5983 |
| evm.TU.scaffold_320.537 | Probable protein phosphatase 2C 4 | 3540 |
| evm.TU.scaffold_206.543 | Heavy metal-associated isoprenylated plant protein 32 | 3382 |
| evm.TU.scaffold_248.387 | Protein downy mildew resistance 6 | 15083 |
| evm.TU.scaffold_5.1954 | Cyclic dof factor 3 | 4425 |
| evm.TU.scaffold_5.1848 | BOI-related E3 ubiquitin-protein ligase 1 | 1503 |
| evm.TU.scaffold_5.1084 | Probable WRKY transcription factor 28 | 2811 |
| evm.TU.scaffold_5.416 | Endoglucanase E1 | 3503 |
| evm.TU.scaffold_384.174 | Trihelix transcription factor GT-1 | 4969 |
| evm.TU.scaffold_461.1152 | Protein cellulose synthase interactive 1 | 2933 |
| evm.TU.scaffold_461.799 | Putative glucuronosyltransferase PGSIP7 | 8486 |
| evm.TU.scaffold_28.831 | Probable 1-deoxy-D-xylulose-5-phosphate synthase | 4602 |
| evm.TU.scaffold_28.1096 | Protein NRT1/ PTR FAMILY 4.3 | 4638 |
| evm.TU.scaffold_35.651 | Protein smax1-like 7 | 7274 |
| evm.TU.scaffold_263.278 | Flavonoid 3'-monooxygenase | 4188 |
| evm.TU.scaffold_46.88 | Cellulose synthase-like protein G2 | 3946 |
| evm.TU.scaffold_375.99 | Serine/threonine-protein kinase STY46 | 7245 |
| evm.TU.scaffold_416.906 | Phosphate transporter PHO1 | 11906 |
| evm.TU.scaffold_196.59 | 3,9-dihydroxypterocarpan 6A-monooxygenase | 3897 |
| evm.TU.scaffold_457.324 | U-box domain-containing protein 16 | 2881 |
| evm.TU.scaffold_419.144 | Probable RNA-binding protein ARP1 | 4482 |
| evm.TU.scaffold_111.321 | Imidazole glycerol phosphate synthase hisHF | 2601 |
| evm.TU.scaffold_111.52 | E3 ubiquitin-protein ligase ORTHRUS 2 | 4165 |
| evm.TU.scaffold_376.221 | Callose synthase 10 | 58771 |
| evm.TU.scaffold_142.305 | Rhamnogalacturonate lyase | 6700 |
| evm.TU.scaffold_376.293 | Galactinol--sucrose galactosyltransferase | 6986 |
| evm.TU.scaffold_36.392 | Trihelix transcription factor GT-2 | 2766 |
| evm.TU.scaffold_42.160 | Probable anion transporter 5 | 9392 |
| evm.TU.scaffold_241.8 | Callose synthase 2 | 25905 |
| evm.TU.scaffold_40.361 | Transcription factor bHLH123 | 3383 |
| evm.TU.scaffold_189.67 | Protein lateral root primordium 1 | 5491 |
| evm.TU.scaffold_40.1100 | Ras-related protein RABE1c | 3968 |
| evm.TU.scaffold_40.1113 | UDP-glucuronic acid decarboxylase 5 | 5373 |
| evm.TU.scaffold_40.1290 | Protein NLP8 | 6122 |
| evm.TU.scaffold_359.1 | Homeobox-leucine zipper protein HOX27 | 4761 |
| evm.TU.scaffold_100.885 | Probable protein phosphatase methylesterase 1 | 6591 |

| | | |
|---|---|---|
| evm.TU.scaffold_461.129 | Cytochrome P450 734A1 | 4805 |
| evm.TU.scaffold_100.1514 | Uncharacterized | 17076 |
| evm.TU.scaffold_264.169 | Caffeoylshikimate esterase | 2942 |
| evm.TU.scaffold_185.165 | Abscisic acid 8'-hydroxylase 1 | 2976 |
| evm.TU.scaffold_416.931 | Cytochrome P450 82A3 | 7123 |
| evm.TU.scaffold_290.445 | Uncharacterized | 9365 |
| evm.TU.scaffold_29.723 | Cytochrome P450 734A1 | 6175 |
| evm.TU.scaffold_162.78 | Bifunctional dihydroflavonol 4-reductase/flavanone 4-reductase | 5558 |
| evm.TU.scaffold_290.274 | Cytochrome P450 82C4 | 5235 |
| evm.TU.scaffold_5.1240 | Bifunctional riboflavin biosynthesis protein RIBA 1 | 5091 |
| evm.TU.scaffold_248.463 | Aldehyde dehydrogenase family 2 member B7 | 4053 |
| evm.TU.scaffold_36.435 | NAC domain-containing protein 82 | 4376 |
| evm.TU.scaffold_205.262 | Phytochrome-associated serine/threonine-protein phosphatase | 6071 |
| evm.TU.scaffold_35.765 | 40S ribosomal protein S4 | 1958 |
| evm.TU.scaffold_222.1754 | S-formylglutathione hydrolase | 4269 |
| evm.TU.scaffold_28.838 | Probable prolyl 4-hydroxylase 4 | 3370 |
| evm.TU.scaffold_5.918 | Zinc finger protein 4 | 5285 |
| evm.TU.scaffold_375.230 | Transketolase | 3814 |
| evm.TU.scaffold_40.1494 | Probable sulfate transporter 3.4 | 5180 |
| evm.TU.scaffold_384.244 | Coilin | 7141 |
| evm.TU.scaffold_36.971 | WEB family protein At1g75720 | 2208 |
| evm.TU.scaffold_222.367 | Putative UDP-glucuronate:xylan alpha-glucuronosyltransferase 4 | 2871 |
| evm.TU.scaffold_241.42 | 26S proteasome non-ATPase regulatory subunit 9 | 5989 |
| evm.TU.scaffold_46.510 | 5'-3' exoribonuclease 4 | 22083 |
| evm.TU.scaffold_44.154 | Eukaryotic translation initiation factor 6-2 | 30582 |

**Table S2** The information of primers of SSR markers in candidate genes

| Locus | SSR | Primer | Product size (bp) | Gene id |
|---|---|---|---|---|
| JXHT002 | (ATA)9 | GGGGATTGCTCATAAGGCGA<br>AGAAGGAAAAAGGGAGCCCA | 244-259 | evm.TU.scaffold_100.36 |
| JXHT004 | (TTC)6 | CAGTTAGGCTGGCGAGAGAG<br>CATCTACAGGGCATCCGGTC | 257-278 | evm.TU.scaffold_100.566 |
| JXHT005 | (CTG)6 | AAGAAGAAGGTGGCGCTTCA<br>TGGACTGTTTCAGGGCCTTC | 284-295 | evm.TU.scaffold_29.376 |
| JXHT010 | (GAA)7 | CGTCAACAAAGGCCACACTG<br>AGAGAGGCCCAACGACTTTG | 225-243 | evm.TU.scaffold_5.1493 |
| JXHT013 | (TAT)6 | GCCGACCAAAGTATCACCCA<br>AGAAGACTGCTCTTGCCGAC | 298-307 | evm.TU.scaffold_5.194 |
| JXHT022 | (CAC)5 | CCACATCCATCACCAAACGC<br>GGGAACTCGGTGTCCATTGA | 279-282 | evm.TU.scaffold_416.207 |
| JXHT025 | (GAA)8 | ACAAATCCACGGCTAGGCAA<br>TCTCCTCTCGTGGTACTCCG | 253-272 | evm.TU.scaffold_97.228 |
| JXHT034 | (TCC)6 | TCACACCTCAACTGAGTCGC<br>TTGGCTAGATGCAGGTGGTG | 130-136 | evm.TU.scaffold_273.148 |
| JXHT051 | (ACA)5 | GGGATTGCCAAAGCATTAGC<br>CCATATCCTATTTCAGGGTG | 171-186 | evm.TU.scaffold_14.215 |
| JXHT062 | (AGG)7 | TCCCTATGGTCGATTTACAC<br>ACGGGTACCATACCCATATC | 169-168 | evm.TU.scaffold_39.962 |
| JXHT066 | (ATT)9 | AAAGGAAGCGAGTACAAACC<br>CTGTAACGTTTCTGACATCAC | 170-185 | evm.TU.scaffold_5.1954 |
| JXHT081 | (TTC)7 | AGGTTTCTTCGAGGCATAGG<br>CAGCAGCTGCTATTGAAACG | 170-185 | evm.TU.scaffold_457.324 |
| JXHT094 | (ATA)7 | TGCAGTGTTCATACCATCGG<br>ACAACCCAACTCAACCGAAG | 174-189 | evm.TU.scaffold_40.1113 |
| JXHT097 | (AAG)14 | ACGTAGCCAACAACAACCCA<br>ACCGAACAGAGCTGGAAAAG | 240-271 | evm.model.scaffold_100.885 |
| JXHT098 | (TAT)7 | ATTGGAGTTGCGCGTTGTTC<br>GGGTTCCTTTCACTGCTGGA | 143-161 | evm.TU.scaffold_29.338 |
| JXHT100 | (AAT)9 | GATGTGGTGCCGTGCTACTC<br>GGCCTGAATCATTAGCCCCA | 333-347 | evm.model.scaffold_461.129 |
| JXHT104 | (TG)12 | AGAGGAAACAGGGGAGCTCT<br>ATGCCCGAAGCCATCAAGAA | 221-239 | evm.model.scaffold_185.165 |
| JXHT105 | (TC)13 | AAAGCCACATCCCTGGTACG<br>GCCGGACAGAATTGGGTGTT | 286-294 | evm.model.scaffold_416.931 |
| JXHT121 | (TA)10 | AGCTCATGTTGCTGAGGGTC<br>CACGCAGCAGTATCCTTTGC | 285-291 | evm.TU.scaffold_248.463 |
| JXHT129 | (TCT)6 | CTCAGAGTCAGACCAACGCA<br>GAAGGTAAACAGCACGTGCC | 246-258 | evm.TU.scaffold_375.230 |
| JXHT130 | (TA)12 | TCCAACAATAGGTGGCAGGT<br>ACCATTGCTAGCCTTGCCAT | 263-271 | evm.TU.scaffold_40.1494 |

| | | | | |
|---|---|---|---|---|
| JXHT133 | (AAT)12 | CTTTCCACCTCCATCGCACT<br>ACCAAGCATGAACTCACCAG | 234-252 | evm.TU.scaffold_384.244 |
| JXHT136 | (ATT)12 | ATTCGAGCTGAACGACGTCG<br>AACATTACAGTGCGCCTTGC | 258-279 | evm.TU.scaffold_241.42 |
| JXHT137 | (TG)24 | GGACACCTAGACCTGGAATA<br>AAGGAGTAATCACCCATAGC | 269-288 | evm.TU.scaffold_46.510 |

**Table S3** Analysis of AMOVA for 11 populations of *Dalbergia odorifera*

| **Source** | ***df*** | **SS** | **MS** | **Variance components** | **Percentage of variation** |
|---|---|---|---|---|---|
| Among populations | 10 | 155972.140 | 15597.214 | 0.000 | 0% |
| Among individuals | 95 | 2226502.360 | 23436.867 | 11411.615 | 95% |
| Within individuals | 106 | 65045.500 | 613.637 | 613.637 | 5% |
| Total | 211 | 2447520.000 | | 12025.252 | 100% |

Note: *df*: degree of freedom; SS: sum of square; MS: mean square.

**Table S4** Pairwise genetic differentiation index ($F_{ST}$) between the 11 populations

| Population | 1 | 2 | 3 | 4 | 5 | 6 | 7 | 8 | 9 | 10 | 11 |
|---|---|---|---|---|---|---|---|---|---|---|---|
| 1 | 0 | | | | | | | | | | |
| 2 | 0.026 | 0 | | | | | | | | | |
| 3 | 0.030 | 0.036 | 0 | | | | | | | | |
| 4 | 0.029 | 0.048 | 0.044 | 0 | | | | | | | |
| 5 | 0.010 | 0.030 | 0.032 | 0.027 | 0 | | | | | | |
| 6 | 0.093 | 0.085 | 0.112 | 0.100 | 0.090 | 0 | | | | | |
| 7 | 0.024 | 0.033 | 0.039 | 0.027 | 0.032 | 0.082 | 0 | | | | |
| 8 | 0.075 | 0.055 | 0.096 | 0.111 | 0.087 | 0.095 | 0.069 | 0 | | | |
| 9 | 0.050 | 0.038 | 0.053 | 0.064 | 0.054 | 0.103 | 0.053 | 0.074 | 0 | | |
| 10 | 0.062 | 0.067 | 0.060 | 0.094 | 0.071 | 0.125 | 0.067 | 0.108 | 0.104 | 0 | |
| 11 | 0.036 | 0.050 | 0.044 | 0.047 | 0.033 | 0.097 | 0.056 | 0.101 | 0.076 | 0.073 | 0 |
